# The Y of cancer sex differences

**DOI:** 10.1002/ctm2.1402

**Published:** 2023-09-07

**Authors:** Jiexi Li, Ronald A. DePinho

**Affiliations:** ^1^ Department of Cancer Biology The University of Texas MD Anderson Cancer Center Houston Texas USA

1

In mammals, the Y chromosome (chrY) determines the male sex and maintains secondary sex characteristics including spermatogenesis. But does the chrY participate in additional biological functions? The answer is a definitive yes. Recent studies have re‐examined the once‐considered “genetic wasteland” chromosome, identifying its role in human cancer biology.

The chrY is largely heterochromatic and extremely gene‐poor.^1^ There are approximately 58 million base pairs on chrY, which is one‐third the size of the X chromosome. Excluding the heterochromatin and pseudogenes, chrY possesses a meager 50 protein‐coding genes. Among them, nearly half are expressed solely in the testis, while another half are expressed ubiquitously or at least in multiple organs,^2^ suggesting physiological functions in somatic tissues. The most well‐known chrY gene is the sex‐determining region Y protein (*SRY*). *SRY* encodes a transcription factor, the expression of which causes testis formation in early development. Many other chrY genes are also involved in spermatogenesis and male fertility, including *DDX3Y*, *PCDH11Y* and *DAZ*.^3^, ^4^ Besides their sexual functions, *DDX3Y* also encodes an MHC‐I male‐specific minor histocompatibility (H‐Y) antigen in leukemic stem cells^5^ and *PCDH11Y* is related to autism.^6^ Two ubiquitously expressed chrY genes, *KDM5D* (also called *JARID1D* or *SMCY*) and *UTY* (*KDM6C*), encode histone lysine demethylases for H3K4 and H3K27, respectively, although UTY possesses minimal histone demethylase activity.^7^

In cancer studies, chrY genes have received less attention compared to genes on the X chromosome, let alone autosomes. Among the chrY protein‐coding genes, only four have been linked to cancers. More importantly, the understudied nature of such genes has resulted in flawed cancer studies. For instance, some chrY gene studies have utilized female cancer cell lines or male cell lines with spontaneous loss of chrY. The misuse of antibodies has also been common, as reflected by the use of antibodies for chrY gene‐encoded proteins showing signals in female cell lines, discrediting these studies. In addition, some studies have injected male cancer cell lines into recipient animals of the opposite sex, overlooking the immunogenic effects of chrY genes. Finally, bioinformatic analyses have also suffered from such indifference to sex, as data analyses unconsciously compare male to female patients, leading to the discovery of false ‘differentially expressed genes’ residing on the chrY.

New studies, including ours, have elevated the role of chrY genes in human disease. Our study, published in Nature,^8^ demonstrated that oncogenic KRAS drives worse outcomes in men with colorectal cancer (CRC) via its regulation of KDM5D. In a CRC mouse model engineered with oncogenic *K* *ras* and conditional null alleles of *A* *pc* and *Tr*p*53* (designated iKAP), male mice showed more metastases and shorter survival compared with females with iKAP CRC. Notably, no sex differences in outcome were observed in KRAS wild‐type (iAP) CRC mice. In iKAP CRC, oncogenic KRAS was shown to upregulate KDM5D via its activation of the transcription factor STAT4. *Kdm5d* deletion in iKAP cancer cells decreased metastasis, while iAP male and female mice with enforced *Kdm5d* transgene expression showed more advanced cancer stages and increased metastasis rate. As a histone demethylase, KDM5D removes H3K4me2/3 at gene promoters and suppresses gene expression; one of the top regulated gene targets was *Amot*, which encodes a tight junction structure protein. Epigenetic repression of *Amot* by KDM5D in metastatic cells resulted in the loss of tight junction integrity and promoted cell migration and invasion; conversely, enforced expression of *Amot* or deletion of *Kdm5d* decreased cell metastatic capability both in vitro and in vivo. In addition, KDM5D interacted with the Sin3‐HDAC complex to remove H3K27ac at super‐enhancers and downregulated genes involved in MHC‐I antigen presentation, *Tap1* and *Tap2*. Deletion of *Kdm5d* sensitized cancer cells to CD8+ T cell‐mediated killing, while *Kdm5d* overexpression diminished CD8+ T cell infiltration at the tumor invasive front, mimicking the effect of KRAS mutation. Together, these findings revealed a genetic mechanism driving sex differences in CRC and identified an actionable therapeutic strategy for metastasis risk reduction for men afflicted with KRAS‐mutant CRC. In addition, two other independent studies published in *Nature* and *Cell* reported the effect of loss of chrY in bladder cancer and across tumor types, respectively. In bladder cancer, loss of the chrY correlated with poor prognoses and CD8+ T cell exhaustion, and in turn, increased responsiveness to anti‐PD‐1 immune checkpoint blockade therapy.^9^ On the other hand, the *Cell* paper's pan‐cancer analysis showed that chrY aneuploidy can either be a passenger or driver event.^10^

The versatile functions of chrY genes in different cancers are fascinating and cry out for further study. For example, KDM5D is clearly a pro‐metastasis gene in CRC but may have protective roles in bladder cancer. This may relate to the distinct genetic mutation profiles in different cancers and differences in cell type biology. For example, KRAS mutation accounts for nearly 50% of CRC, yet is encountered in less than 30% of bladder and urinary tract cancers. Other common mutations in CRC are APC and p53 loss‐of‐function mutations, whereas the top mutations in bladder cancer are found in the *FGFR3*, *PIK3CA*, *KDM6A* and *TP53* genes. In addition, the timing of mutation emergence and clonal evolution trajectories also vary. As a result, various growth signaling pathways are activated in cancer cells, affecting transcriptomic and epigenomic profiles, and generating distinct growth advantages and vulnerabilities for cells of different cancer types. Moreover, the tumor microenvironment, exposure to carcinogens, and microbiota of different cancers also contribute to the ‘friend‐or‐foe’ roles of genes.

Context‐specific differences aside, these new studies highlight the important roles of chrY genes in key cancer hallmarks and emphasize that a patient's sex is an imperative factor in cancer prognosis and treatment. Based on our findings in KRAS‐mutant CRC, we suggest that men should undergo a higher level of surveillance for recurrence (particularly for patients with surgically resected stage II tumors) and be considered candidates for adjuvant therapy. Furthermore, the development of KDM5D inhibitors may offer an additional therapeutic option for men, alone or together with immune therapy. Altogether, we now have a better understanding of ‘why’ men experience worse outcomes in certain cancers.

## CONFLICT OF INTEREST STATEMENT

Ronald A. DePinho holds equity as a former advisor and/or director of Tvardi Therapeutics, Asylia Therapeutics, Stellanova Therapeutics, and Sporos Bioventures. Jiexi Li declares no conflict of interest.

## FUNDING INFORMATION

Jiexi Li was supported by the Cancer Prevention and Research Institute of Texas (CPRIT) Research Training Program (RP210028). Ronald A. DePinho was supported by NIH (National Cancer Institute) R01 CA231360, CPRIT (RP220364), the MD Anderson SPORE in Gastrointestinal Cancer‐DRP Award, and the Harry Graves Burkhart III Distinguished University Chair in Cancer Research.
